# Influence of introducing a story stem in an interactive play context on maternal and their four-year-old children’s use of mental state language

**DOI:** 10.1371/journal.pone.0311237

**Published:** 2024-09-30

**Authors:** Mette Skovgaard Væver, Camilla Overbye Roos, Johanne Smith-Nielsen, Ida Egmose, Katrine Isabella Wendelboe, Anne Christine Stuart

**Affiliations:** Department of Psychology, University of Copenhagen, København, Denmark; Atlantic Technological University, IRELAND

## Abstract

Maternal and child mental state language is associated with improved socioemotional and cognitive child development. This study examined if introducing a story stem (a narrative playing out socioemotional conflicts) in a play situation facilitated maternal and child mental state language compared to a free-play (baseline) situation, and if mothers and children with low baseline mental state language profited more from the story stem situation. Participants were 101 four-year-old children and their mothers. Maternal and child mental state language correlated and providing the story stem increased both maternal and child mental state language. Providing a story stem increased mental state language more for mothers and children groups with lowest mental state language at baseline compared to a high mental state language group. The results indicate a validation of the use of story stems to increase maternal and child mental state language within a typical population.

## Introduction

Parental and child mental state language (MSL) is defined as mental state references, i.e., references to cognitions (e.g., think/know), emotions (e.g., happy/upset) or desires (e.g., want/like), occurring during an interaction [1]. The mental state references may reflect the parent’s or the child’s mental states during the interaction, but they could also be in relation to characters in a book or figures used in a pretend play setting. Several studies have shown that parental and child MSL is key for children’s development of understanding their own and other’s minds [2–6]. Given the importance of MSL for child development, it is essential to examine whether it is possible to promote parents’ and children’s use of MSL. Therefore, we examine whether providing mothers and typically developing 4-year-old children with a directive and defined story stem increases their use of MSL compared to a non-directive free-play (baseline) situation.

### Child mental state language is an indicator of social understanding

The caregiver-child relationship provides an important context for the child to learn about mental states, which in turn is vital for the child’s socioemotional and cognitive understanding [2]. Between the second and third year, children begin to use MSL with increasing frequency and complexity [2, 7]. In the third year, children begin to understand that other people can have beliefs, desires, and intentions different from their own, which are important milestones in the development of Theory of Mind (ToM: The understanding that unobservable mental states, such as thoughts, beliefs, and emotions, underlie most human behavior; [8]) and social understanding [2]. Children’s own use of MSL indicates their understanding of mental states underlying behavior [4]. This is supported by results of children’s own MSL correlating with their emotional understanding [9], and that children who frequently use MSL perform better on ToM (false belief) tasks [10] and have better social interactional skills [11, 12]. Based on these findings, child MSL may be considered a solid indicator of their social understanding [3].

### Parental mentalization and mental state language is important for child development

Several studies have shown the importance of parental mentalization, i.e., the parents’ ability to perceive their children as individuals with their own minds who are capable of intentional behavior [13], as well as parental MSL for the parent-child relationship quality (e.g., attachment) and child long-term socioemotional and cognitive development [1, 14–21]. For example, Razuri et al. [2] examined the longitudinal roles of infant-mother attachment quality and maternal MSL for child MSL development from 12 to 52 months. They showed that child MSL increased over time, that there were differences between the type of mental state words and patterns of mental state words used by mothers in secure versus insecure dyads, and that the quality of child attachment appeared to exert a consistent positive influence on child MSL over time. Further, a recent meta-analysis found, in a sample of 2,298 children below seven years, small significant effect sizes for parental MSL on child emotion understanding and ToM (false beliefs understanding) even when controlling for children’s age and language [5]. All of this provides an indication that the caregiver-child relationship provides an ideal context for the child to learn about and develop and understanding of mental states.

### Context and mental state language

It is debated whether parental MSL in certain contexts has a stronger effect on child development than in other contexts. For instance, a meta-analysis found that parental MSL during free play, toy play, and book reading with their child showed stronger associations with child social understanding than parental MSL during reminiscing [5]. In contrast, another meta-analysis did not find the setting to be a significant moderator of the effect of parental MSL on child social understanding, but reminiscing was not included [22]. Studies have shown that interactive contexts involving storytelling (e.g., book reading) increase parental use of MSL compared to other contexts (e.g., free play) [23], and that it is the act of narrating in itself, and not necessarily the stimuli used in the storytelling (e.g., book/figures), that seem to promote the use of MSL in this context [24]. Farkas et al. [23] compared maternal MSL in a storytelling versus a free-play context in a sample of 91 mothers and their 10-15-months-old children. They also examined how maternal MSL in the two contexts related to child language and socio-emotional outcomes at 28–33 months. They found that providing parents with a story stem promoted more maternal use of MSL, and maternal MSL in both interactive contexts was related to child language and socioemotional skills.

Fewer studies have examined how different interactive contexts affect child use of MSL. One of the mechanisms hypothesized to explain the effect of parental MSL on child social understanding is the use of highlighting and contrasting individual’s perspectives (e.g., “I like” or “She thinks”) during conversations. By highlighting this contrast in conversations, the child is made aware and realizes that everyone has their own representation of the same experience and feelings, desires, beliefs are subjective [25]. Several studies have shown positive correlations between parental and child MSL [2–5, 23, 26–28]. Tompkins et al. [6] analyzed contingencies between maternal and child mental state utterances and showed that maternal MSL elicits child MSL. Nevertheless, it is important to also directly examine whether different interaction settings affect child MSL. To the best of our knowledge, only one previous study examined the effect of different interactive contexts on child MSL. Roby and Scott [29] found that 55 mothers and their 3-year-olds used more MSL during picture book sharing compared to free play. However, more studies are needed to consolidate these findings.

### Pretend play and mental state language

A venue for promoting both mother and child MSL may be by introducing story stems in pretend play scenarios. Pretend play is an overall term for play activities that include fantasy, sociodramatic, or role-play, and it increases around the ages of 3–4 years [30]. Leslie [31] and Hughes and Dunn [12] argued that children who engage in pretend play frequently are more likely to use MSL in conversations, suggesting that pretend play elaborates the children’s mental state lexicon. In cases of clinical child assessment (e.g., Story Stem Assessment Profile; [32]), the introduction of a story stem in an interactive play situation is known to be an ideal context for the child to engage in pretend play. It involves the child being presented with the beginning of a story, i.e., a stem, which the test administrator acts out using a standard set of toys, typically family or animal figures. The story stem consists of an everyday problem with a controlled degree of conflict or distress that is intended to induce emotional arousal in the child. When the administrator stops the story stem, the child is asked to continue and play out the story with the figures [33]. Since the story stem combines storytelling and pretend play in a structured and controlled setting, story stems could be considered optimal for promoting both parental and child MSL. In the study by Farkas et al. [23], vignettes and a set of toys in the mother-child interaction were introduced, and the authors found that this promoted more maternal MSL. However, they did not address the question whether this also promoted child MSL.

### The present study

Based on the reviewed literature, the aim of the current study was to examine if providing mothers of 4-year-old children with a story stem to initiate play would promote more maternal *and* child MSL when compared to a free-play situation (baseline). Based on previous findings that maternal MSL is associated with child MSL, our first aim was to replicate this and to investigate the hypothesis that maternal and child MSL correlates in both the story stem and the free-play situations in this study. Based on the previous findings that storytelling promotes maternal MSL, we further hypothesized that adding a story stem would increase not only maternal but also child MSL during story stem play compared to a free play situation. Finally, we wanted to explore whether there were differential effects of introducing the story stem to mothers and children’s MSL depending on their levels of MSL in the free play context (baseline). To the best of our knowledge, no previous studies have examined whether a story stem impacts MSL differentially, but Roby and Scott [29] found that there were differential effects of the task context’s effect on MSL depending on ethnicity. Further, Symons et al. [10] found that parents who already engage their children in conversations about the character’s feelings, thoughts, etc., also made it a habit for the children to converse about their own mind. These findings tentatively indicate that a story stem may not have the same impact across different groups. We explore whether mothers and children with little to no MSL at baseline compared to mothers and children with more MSL benefits differentially from the story stem.

## Methods

### Sample

Mother-child dyads (*N* = 101) were recruited from parenting groups identified via advertisements on the center’s social media platforms in and around Copenhagen, Denmark. Data was collected from December 11, 2015, to December 14, 2016. The research received approval from the Institutional Ethical Review Board of the Department of Psychology at the University of Copenhagen (Approval number: 2015/11). Inclusion criteria were first-born, Danish-speaking, typical developing children with mentally and physically well mothers. Table 1 shows sample characteristics. In general, the mothers were well-resourced (i.e., well-educated, employed, living with a partner). Child verbal ability was assessed using Verbal Comprehension Index (Wechsler Preschool and Primary Scale of Intelligence™ –Fourth Edition, WPPSI™–IV, [34]), and the children scored over one standard deviation of the norm.

**Table 1:** Sample characteristics (*N* = 101).

Maternal age, *M* (*SD*)	34.62	(4.31)
Maternal education, *n* (%)		
9 years (ISCED level 2)	1	(1.0)
12–13 years (ISCED level 3)	12	(11.9)
14–15 years (ISCED level 5 & 6)	57	(56.4)
17 years (ISCED level 7)	27	(26.7)
20 years (ISCED level 8)	2	(2.0)
Maternal education in years, *M* (*SD*)	15.38	(1.63)
Mother marital status, *n* (%)		
Living alone	14	(13.9)
Living with partner/married	87	(86.1)
Maternal employment status, *n* (%)		
Employed	75	(75.8)
Unemployed	24	(24.2)
Child age, *M* (*SD*)	4.52	(0.11)
Child sex, *n* (%)		
Male	56	(55.4)
Female	45	(44.6)
Child verbal ability, *M* (*SD*)	113.37	(19.34)

*Note*. ISCED = International Standard Classification of Education by UNESCO, 2011

We used the study by Farkas et al. [23] to calculate effect sizes for a power calculation. While they did not report any effect sizes, based on their descriptive statistics at both 12 and 30 months, they found large effect size in the difference of MSL for the free-play versus storytelling contexts. Thus, for our study, we would need a sample size of 31 to detect a large effect size (Cohen’s *f*^*2*^ = .35) for a linear regression with two predictors with a significance level of .05 and a power of 80%. As we also wanted to have enough power to detect a medium effect size (Cohen’s *f*^2^ = .15), we recruited 100 participants.

### Procedure

All parents gave written informed consent prior to enrollment. Each mother-child pair participated in a session at a university laboratory setting, lasting approximately two hours and consisting of three parts: (1) play session with mother and child; (2) child test session with researcher; and (3) maternal questionnaire session with a research assistant. During the child test session, the mother completed questionnaires in a separate room. The play session took place before the tests and questionnaires. This was done to ensure the children’s comfort and sense of security. Having the play session with their mothers first allowed them to get used to the laboratory setting before they were asked to be alone with the researcher during the test situations.

**Fig 1:**
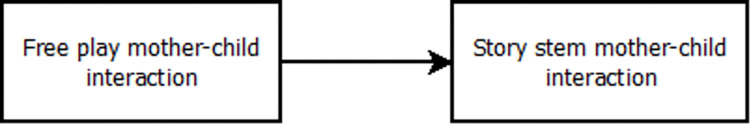
Graphic of the procedure in the current study.

The play session consisted of seven successive different play situations each of five minutes duration. The play session took place in a dedicated observational room and was monitored by researchers through a one-way mirror and recorded via three cameras installed at different angles. The mother was given instructions beforehand and offered the opportunity to ask questions. The researcher signaled the beginning of each set-up by knocking on the one-way mirror, after which the mother opened a matching envelope containing a short and simple instruction for the mother. For this study, data from play situation two (interactive free-play mother and child) and four (interactive story stem mother and child) was applied and the remaining situations are therefore not described here. For a description of all play episodes, see Stuart et al. [35]. The procedure for the current study is depicted in Fig 1. The same set of toys were provided for both interactive situations used in this study. The toys were LEGO DUPLO™ and included a playhouse with furniture (four chairs, a table, a kitchen, 2 beds, and a toilet); figures (a man, a woman, a boy, a girl, a policeman and thief, a princess, and two knights); various animals (a cow, a horse, and a crocodile); a motorcycle, a fire truck, 24 lose bricks, a slide, money, cake, a suitcase, a sun/rain-brick.

In the mother-child free play situation, the mother was invited to engage in free play with her child with the instruction, *“Please engage in free-play with your child*. *Just do what you always do or play as you normally play with your child*.*”* In the situation where the story stem was introduced, the mother was instructed to read a story stem aloud to the child prior to commencing interactive play with the instruction, *“Please read out loud the story in the envelope*. *The story is special in the way that it is unfinished*, *and you finish by asking “What happens next*?*” and your child and you must complete the story using ideas that you create*.*”* The story stem reads, *“The family is about to celebrate the birthday of the boy/girl (child´s choice) but the boy/girl falls on the slide and hurts him/herself*. *What happens next*?*”*

### Measures

#### Demographic information

Demographic information was collected through questionnaires during the laboratory visit. This data was collected to be able to test whether any of the variables should be included in the analysis as control variables, as for example maternal age, maternal educational level, and child gender may have the potential for affecting maternal MSL. Several parental abilities are known to be strongly correlated with educational level, which is also the case for maternal mentalizing and MSL [29, 36].

#### Child verbal abilities

Child verbal ability was assessed with Wechsler Preschool and Primary Scale of Intelligence™ –Fourth Edition (WPPSI™–IV, [34]). For this study, the Verbal IQ scores, i.e., the Verbal Comprehension Index (VCI), were used. VCI measures the child’s acquired knowledge, verbal reasoning and comprehension skills, and ability to pay attention to verbal stimuli as it is presented. Child verbal abilities data was collected to be able to test for whether this should be included in the analysis as a control variable, as it may potentially affect child MSL [37].

#### Mental state language (MSL)

Maternal and child mental state language (MSL) was coded from the video recordings of the five minutes free-play and the five minutes story stem situations. MSL has often been measured in tasks that are designed to elicit MSL and as such may provide inflated results [38]. To avoid an inflated measure of MSL for the baseline measure in the free play session, no instructions were given to the mothers other than to play as they normally would with their child to allow for as naturally occurring instances of MSL as possible. Following previous research [23], we used categories adapted from the mind-mindedness measure (Meins & Fernyhough [Unpublished]) to include both maternal and child MSL about not only the child’s inner life, thoughts, and feelings, but also those of the mother and the characters in the play situation. The child’s and mother’s MSL was coded into one of three mutually exclusive categories: 1) Desire and Preferences, e.g., like, don’t like, prefer, love, hate; 2) Cognitions, e.g., think, decide, know, remember, focus, expect; and 3) Emotions, e.g., happy, sad, scared, shy, confused, excited, upset. When coding for MSL when speaking on behalf of the play figures, all utterances that were obviously meant to be dialogue said or thought by the play figures were coded in accordance with the criteria for mind-related comments in the mind-mindedness coding manual (Meins & Fernyhough [Unpublished]). All maternal and child utterances of MSL were tallied during the two five-minute video recordings and coded as MSL, excluding immediate repetitions (e.g., “I want…I want…”). As the recordings in this study were timed and each had a duration of five minutes, we used frequencies to assess maternal and child MSL. Previous studies have calculated both frequencies of MSL and proportion of MSL to total spoken language and found identical patterns of results [39, 40]. Likewise, a recent meta-analysis suggests that frequency measures of mental state talk may be a more sensitive predictor of children’s false-belief understanding compared to proportion of mental state talk [5]. A trained research assistant coded MSL and a randomly selected subset (20%) was coded independently by another trained coder to determine interrater reliability. The *average measures* intraclass correlation coefficients (ICC) for the free play session, using a two‐way mixed‐effects model with absolute agreement, were ICC = .994 (*p* < .001; 95% CI [.992; .995]), indicating excellent reliability. For the story stem play-context, the ICC also indicated excellent reliability (ICC = .978, *p* < .001, 95% CI [.971; 984]).

### Analysis strategy

To assess whether demographic information and child verbal IQ should be used as covariates, we used zero-order correlations between child and maternal MSL in the two situations and maternal age and child VCI, and we used Poisson loglinear models for maternal educational level and child gender and MSL scores. Maternal educational level was collapsed into three levels which related to lower education (ISCED level 2 and 3), secondary educational level (ISCED level 5 and 6), and high education (ISCED level 7 and 8). Maternal educational level was significantly associated with both maternal and child MSL scores in the free-play and story stem interactions (all *p*s < .05), and child gender was significantly associated with child MSL in the free play situation (*p* = .006). Maternal educational level and child gender were thus included in all subsequent analyses as covariates. No other sociodemographic information or child verbal IQ were significantly associated with MSL in either the free play or story stem interaction (all *p*s ≥ .124) and were thus not included as covariates in any of the main analyses.

To investigate our first hypothesis that maternal and child MSL correlates in both a free-play and story stem situation, we used Spearman correlations. We interpreted the effect sizes as small (*r* = .1), medium (*r* = .3), or high (*r* = .5) [41]. The second hypothesis, i.e., that maternal MSL and child MSL would increase with story stem play, was investigated using generalized estimating equations (GEE) with a first-order autoregressive (AR(1)) covariance structure. Since MSL is a count variable, we used a Poisson loglinear model to analyze it. First, we investigated if there was a significant increase in maternal and child MSL from free play to a story stem situation, separately. Second, we investigated whether an increase in maternal MSL was associated with an increase in child MSL. Finally, for our explorative analyses, we also used the above-described GEEs to investigate whether MSL would increase in the story stem play situation more for mothers and children with little to no MSL in the free play situation (baseline). As no established cut-off scores for low and high MSL exist, we divided mothers into two groups based on their median free play (baseline) MSL scores, i.e., low MSL (0–1 words) and high MSL (≥ 2 words). The same was done for child MSL, with low child MSL being 0–1 words and high MSL being ≥ 2 words. Overall, a small amount of mental state talk is typically observed in studies of maternal and child MSL (Tompkins et al., 2018). We chose to divide by the median score to get as equal group sizes as possible when we do not have any official cut-off values for what is termed high and low MSL. A significant interaction between MSL group and play session would indicate support for our explorative hypothesis. All analyses were conducted in SPSS Statistics 28 (IBM, Chicago, IL), and all reported *p*-values are two-tailed and evaluated at a significance level of .05.

## Results

Table 2 shows descriptive statistics for maternal and child MSL. In general, we see a positive increase in both scores from a free play to a story stem play situation. Further, Table 2 shows correlation coefficients for the variables. All the correlation coefficients are medium or high and positive, indicating that if maternal MSL increases, child MSL increases as well in both play situations. Our first hypothesis was thus supported.

**Table 2:** Descriptive statistics and Spearman correlation coefficients (*N* = 101).

	1.	2.	3.	4.	M	SD	Range
1. Free play child MSL	-	.64*	.43*	.39*	4.71	6.82	0–30
2. Free play maternal MSL		-	.38*	.35*	5.50	7.85	0–39
3. Story stem child MSL			-	.82*	9.14	8.27	0–34
4. Story stem maternal MSL				-	12.01	9.52	0–42

*Note*. MSL = Mental state language; *p < .001

GEEs showed that child MSL increased significantly from free play to story stem play (*b* = .66, Wald χ^2^(1) = 28.92, *p* < .001, 95% CI [0.42; 0.90]). Maternal MSL also increased significantly (*b* = 0.77, Wald χ^2^(1) = 36.43, *p* < .001, 95% CI [0.52; 1.03]). This indicated that the amount of MSL for both mothers and children increased in the story stem play situation. A Poisson loglinear regression analysis showed a positive relationship between the development of maternal MSL and development in child MSL (*b* = 0.07, Wald χ^2^(1) = 343.49, *p* < .001, 95% CI [0.06; 0.08]), indicating that if the mother’s MSL increased by 1 word when adding a story stem, her child’s MSL also increased by 0.07 words. Our second hypothesis was thus supported.

**Fig 2:**
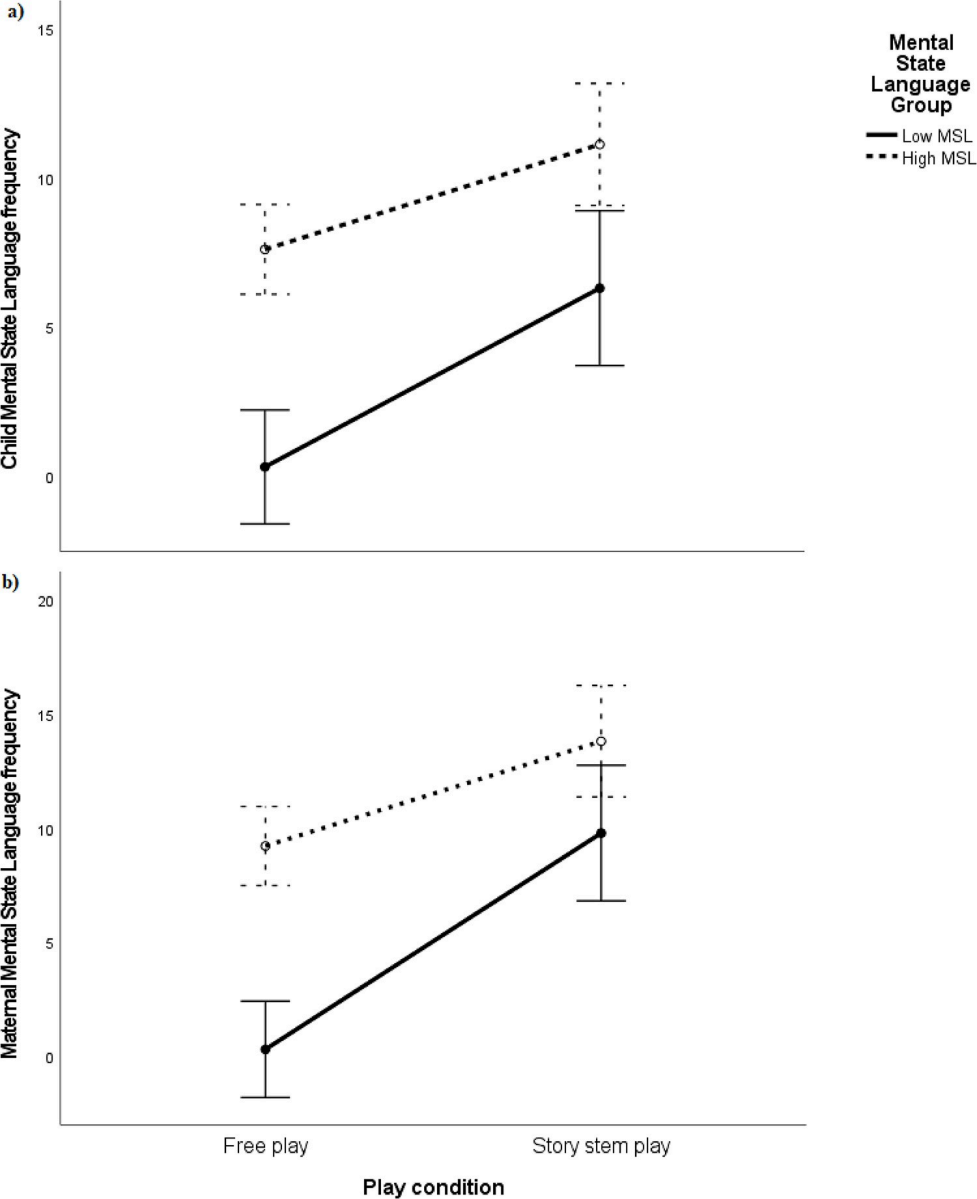
Interaction mean plot of Mental State Language (MSL) scores for children (a) and mothers (b) with high and low MSL in the two play situations for children and mothers with high and low MSL free play (baseline) scores. Error bars are 95% confidence intervals.

Table 3 shows descriptive statistics for the two groups (high/low MSL) for mothers and children. In general, we see a bigger increase for the two low baseline MSL groups compared to the high MSL groups. For maternal MSL, a Poisson loglinear model showed that there was a significant interaction between maternal low/high MSL group and play session with mothers in the high MSL groups profited less from the story stem compared to the low maternal MSL group (*b* = -2.81, Wald χ^2^(1) = 130.10, *p* < .001, 95% CI [-3.29; -2.33]). For child MSL, we found the same pattern that the children in the high MSL group said less MSL in the story stem play situation compared to the low child MSL group (*b* = -2.68, Wald χ^2^(1) = 67.67, *p* < .001, 95% CI [-3.32; -2.04]). These interactions are also depicted in Fig 2. We, thus, found an indication of support for our explorative hypothesis.

**Table 3:** Descriptive statistics for high/low MSL groups.

	Difference in maternal MSL	
	M	SD
Low maternal MSL (*n* = 42)	9.26	7.95
High maternal MSL (*n* = 59)	3.61	7.58
	Difference in child MSL	
	M	SD
Low child MSL (*n* = 39)	5.85	6.00
High child MSL (*n* = 62)	3.53	8.09

*Note*. MSL = Mental state language

## Discussion

In the present study, we investigated the associations between maternal and child MSL in two different mother-child play situations–a free-play and a play situation where a story stem was introduced. Further, we investigated whether introducing a story stem could promote both maternal and child MSL and explored whether those dyads who had a low amount of MSL at baseline (the free play situation) benefitted more from the story stem.

As there were significant associations between maternal and child MSL, our first hypothesis was supported. The correlations between child and maternal MSL were high in both play situations, indicating that if one of them had a high amount of MSL, then the other also did. This is in line with previous studies that consistently report the same positive association between maternal and child MSL [2–5, 23, 26–28]. There were also significant correlations between free-play and story stem play MSL for both the mothers and children. These positive, medium correlations indicate that although different contexts may affect the amount of MSL, there is also a moderate degree of stability of MSL.

Supporting our second hypothesis, we found a significant increase in MSL in the story stem play situation compared to free play for both mothers and children. Further, the Poisson loglinear regression showed that if the mother’s MSL improved in the story stem situation, then so did her child’s. These findings are in line with previous research findings that maternal and child MSL increased during a storytelling interaction compared to free play [29]. Extending previous studies, which have examined the effect of picture-book-reading (e.g., [29]) or maternal storytelling (e.g., [23]) on MSL, we examined whether introducing a story stem during a play scenario would elicit MSL in mothers *and* children. The story stem used in the present study consisted of an everyday problem that included a little distress, which was intended to induce emotional arousal in the child and to support the mother and child play out the end of the story with the figures. Since the story stem combines storytelling and pretend play in a structured and controlled setting, story stems could be considered optimal for promoting both parental and child MSL. Symons and colleagues [10] argue that parents who regularly ask their children about book characters’ thoughts/feelings encourage the children to also ask such questions about themselves, thus developing a habit of conversing about the mind. As a story stem is quite similarly structured, in that it sets the stage for a narrative with characters that the child is asked to unfold, it seems to be a useful and simple venue for promoting both maternal and child MSL. The use of story stems to promote MSL and dialogues about the mind could also potentially be implemented in a daycare setting with day care providers supporting pretend play. A recent study suggests that providing roleplay material to children stimulates them to engage in social pretend play, but that adults’ play support increases the quality of social pretend play [42], and this could potentially be assisted by using a story stem. Research on adult participation in play generally find a significant increase in the child’s pretend play quality as long as the adult is actively engaged in, contributes to, and structures the play compared to passive observation or very little participation (e.g., [43–45]). We have in multiple studies found that using a story stem increases the child’s play complexity compared to other forms for interactive mother-child play scenarios [35, 46]. A story stem may thus be a way of ensuring the active participation needed to lift pretend play as well as MSL.

Finally, we explored whether introducing a story stem would promote MSL more for mothers and children with low MSL in the free-play situation and found significant results. These results indicate that mothers and children who exhibited none-to-low amounts of MSL in the free-play situations improved their amount of MSL more when introducing a story stem compared to the group of mother and children with higher amounts of MSL in the free-play situation. To the best of our knowledge, only Roby and Scott [29] have examined whether different contexts have differential effects on promoting MSL in different groups. They found that there was a significant interaction between ethnicity and task, meaning that there were differential effects in how beneficial the context was to the mother and children’s MSL depending on the mother’s ethnicity. However, their finding is based on a between-group level, whereas our analysis is within-subject based, indicating that the differential effect in the context also depends on the participant’s natural/baseline levels of MSL. This would also be somewhat in line with Symons and colleagues’ [10] findings that parents who already naturally ask their children about character’s state-of-mind develops a habit of conversing about the mind, meaning that these dyads where it is already a habit do not improve further by introducing a story stem. Taken together, our results indicate that a story stem of five minutes can improve MSL for both mothers and children. Future research should investigate whether a longer play intervention with story stems yields the same results longitudinally, indicating that the improvement in MSL is stable in mother-child dyads with a low amount of mental state talk.

Our study has some limitations that are important to consider. First, these findings may not be generalizable due to sample characteristics. The sample was well-resourced and low-risk. Maternal mentalizing capacities may differ in at-risk mothers [47] which may also affect their knowledge of the importance and the ability of increasing their child’s MSL. Second, we did not randomize the temporal order of the play situations, and hence, the story stem always came after the free-play situations. Thus, we cannot conclude that increased MSL is solely due to the story stem; it may be that the mothers and children felt more comfortable 15 minutes into the play sessions. The play sessions have a natural progression as each session builds on the previous one by adding one new element, and we decided not to randomize to rule out any carry-over effects. However, future research should consider a cross-over design to further investigate the effect of a story stem on MSL as well as whether there are any carry-over effects in that mothers and children use the story stem approach when engaging in free-play interactions. Third, the study only focused on maternal MSL and not paternal MSL. We cannot know whether introducing a story stem would increase paternal MSL and its association with child MSL. Mothers have been found to talk more about emotions with their children than fathers [48, 49], and it would thus be interesting to investigate whether gender differences affect the association between parental and child MLS.

## Conclusion

In conclusion, this study furthers the evidence of an association between maternal and child MSL as well as indicates the introduction of a story stem to increase MSL of both mothers and children. A story stem is potentially a very simple and inexpensive way of supporting caregivers and children increasing their mental state vocabulary and mentalizing abilities. It may be a promising way of aiding parents and children with limited mentalizing skills to increase their MSL and mentalization capacity in general. Future research should investigate whether increasing MSL in play situations will have a spillover effect to other conversation context and have any longer lasting effects on mentalizing and theory of mind development.
